# High burden of STI and HIV in male sex workers working as internet escorts for men in an observational study: a hidden key population compared with female sex workers and other men who have sex with men

**DOI:** 10.1186/s12879-015-1045-2

**Published:** 2015-07-29

**Authors:** Amanja Verhaegh-Haasnoot, Nicole H. T. M. Dukers-Muijrers, Christian J. P. A. Hoebe

**Affiliations:** Department of Sexual Health, Infectious Diseases and Environmental Health, South Limburg Public Health Service, 6160 HA Geleen, The Netherlands; Department of Medical Microbiology, School of Public Health and Primary Care (CAPHRI), Maastricht University Medical Center (MUMC+), 6202 AZ Maastricht, The Netherlands

**Keywords:** Male sex workers, Sexually transmitted infections, HIV infection, Men who have sex with men, Female sex workers, Key populations, Public health policy

## Abstract

**Background:**

Male sex work in the western countries has changed, including now a subculture of male sex workers who have paid sex with men arranged for via the internet. The men involved in this subculture do not easily identify themselves as sex workers nor as homosexual, and are therefore missed by regular health care and public health interventions. These male sex workers may form a hidden key population for sexually transmitted infections (STIs) and HIV, bridging towards other persons outside this context.

**Methods:**

This clinic-based observational study included consultations by male sex workers (*n* = 212), female sex workers (*n* = 801) and in men having sex with men who did not report being paid for sexual contacts (MSM, *n* = 2703) who received STI and HIV testing and counselling at our clinic during the study period. In this study we compare the consultations in male sex workers to those in in female sex workers and MSM.

Demographic characteristics and sexual behaviour of the male sex workers, female sex workers and MSM were compared using chi-square tests and non-parametric tests. Using univariate and multivariate regression analyses, determinants for STI positivity in male sex workers were evaluated.

**Results:**

Male sex workers tested positive for STI (including HIV) in 40 % of the consultations; female sex workers and MSM respectively in 9 and 14 % of the consultations. A new HIV infection was found in 8 % of the consultations of male sex workers. Male sex workers were a young population of migrant sex workers from Eastern Europe. They reported more often to also have sex contacts with women and other sex workers. Male sex workers are at a higher risk for one or more new STI than female sex workers and other MSM, even after correction for age, ethnicity, known HIV positivity and behavioural variables.

**Conclusions:**

Male sex workers form a hidden key population that impacts the transmission of STI and HIV within the MSM population and, possibly, to the heterosexual population. They require specific targeted interventions. Although targeting male sex workers is labour intensive it is feasible and important to reduce STI transmission.

## Background

Sex work has long been present in society. Over time the settings and circumstances change, creating new risks for sex workers and their clients. Men who sell sex to men are a diverse population across countries [1]. In recent years the setting of male sex work in western countries has shifted towards a subculture of men having paid sexual contacts with men who they encounter via the internet [2]. These male sex workers are often home-based internet escorts who do not self-identify as homosexual nor refer to themselves as sex workers [3].

The male sex workers visiting our sexually transmitted infection (STI) clinic often don’t refer to their own activities as sex work, but as being involved in “escort”. Their sexual behaviour includes paid sexual contacts with men, often without using a condom. The male sex workers often do not refer to themselves as homosexual [4–9], nor do they refer to their activities as sex work. and, even though contacts are made online - these male sex workers tend to keep their activities hidden from public attention.

In the Netherlands, selling sexual services is a legitimate economic activity. Many of the sex workers in the Netherlands and neighbouring countries are at the moment originating from Eastern European countries like Bulgaria and Romania [10]. Migrant workers from these countries often are for a short period of time in the country where they work and are concerned with making money [11]. Despite the legal status of sex work in the Netherlands, there were during the study period restrictions for employment in sex work of people originating from new European Union member states, like Bulgaria and Romania (who joined the European Union in 2007). Citizens from these countries can legally stay in the Netherlands and work as independent escort, but were not allowed to be employed at a sex venue. There are also difficulties for inhabitants of these countries to access the medical care system, due to lack of knowledge and legal and organizational barriers [11]. Therefore this hard-to-reach population doesn’t come in contact with regular health care and also remains hidden from traditional public health activities. As a consequence, these men are often neglected in STI control prevention programmes. When they are infected with an STI or human immunodeficiency virus (HIV), untreated male sex workers continue playing a part in the chain of transmission.

Most research on male sex workers was done in the late 1990s and early 2000s and described male sex workers involved in street sex work [5–7, 12–14]. In 2013 ten European countries reported a HIV prevalence among male sex workers of 8.9 % to the UN General Assembly Special Session [15]. In some studies sex workers were described who were involved in home-based sex work or escort services, but their sex work was often combined with sex work on the streets or in sex venues for example sex clubs [5–7, 12–14]. A recent study in England described male sex workers who have sex with men or women as a risk group, but it gave no insight into underlying risk factors [16]. A similar phenomenon of men having sex with other men for money is known in other countries around the world. Especially in China, studies of the male sex workers subculture have been conducted [17]. However, the general culture and gay culture in particular in Asian countries differs from that in western countries: the different behaviours (including sexual behaviour), attitudes and settings (including risk settings) make a direct translation of the research findings not applicable. In the Netherlands there is a culture of tolerance towards homosexuality, resulting in laws against gay discrimination and same-sex couples having been able to adopt children since 2001. Despite this, men having sex with men still face inequalities in the Netherlands [18]. The situation differs from for example China, where homosexuality and same-sex relationships often conflict with Chinese cultural traditions and face societal disapproval [19].

Targeting male sex workers is labour intensive and requires investing time and resources into building a relationship based on trust, by means of fieldwork and outreach activities. From 2009 on we initiated regional outreach activities directed at male sex workers by contacting potential male sex workers through their internet escort work advertisements. Trained public health nurses were involved in fieldwork targeting male sex workers: they contacted the male sex workers via the advertisements, or by contacting them via other (male) sex workers in their (temporary) homes. This was also the location where most of the male sex workers’ sex activities took place. The main goal was to engage the men in STI and HIV testing at location or at our STI clinic, provide counselling on HIV and STI prevention and provide treatment when tested positive. There are already many targeted interventions for established high risk groups, such as female sex workers and men having sex with men who are not engaged in sex work (MSM). Only few studies addressed the risk of STI and HIV transmission in male sex workers, demonstrating a consistent higher prevalence of HIV and STIs [1]. Similarities and disparities between internet-based male sex workers, MSM and female sex workers are unknown. One objective of this study is to assess the positivity rate of STI and HIV in internet-based male sex workers, female sex workers and MSM. The positivity rate describes the number of cases testing positive as a proportion of the number submitted for testing We also aim to assess whether and to what extend underlying risk factors influence significant differences between male sex workers, female sex workers and MSM. Assessing these determinants for STI positivity between male sex workers female sex workers and other MSM, may help to inform targeted interventions.

## Methods

### Study design

This clinic-based observational study included data on male sex workers, MSM and female sex workers who received HIV and STI testing and counselling at the STI clinic run by the Public Health Service South Limburg, in the Netherlands from January 2009 to May 2012. Male sex workers, MSM and female sex workers were pro-actively targeted in outreach activities directed at these different groups. As male sex workers are a population largely hidden to society, probabilistic sampling was not feasible. Initial contacts with the female and male sex workers were made online. After the initial contacts were made, especially in the group of male sex workers, other sex workers were included via snowball sampling or when encountered during testing at outreach locations. Most male sex workers were tested during outreach activities aimed at the ‘home’ environments where their sex activities took place. These home environments were often houses where a group of male sex workers temporarily resided and also offered their sexual services. Outreach activities targeted at female sex workers took place at known sex venues, while outreach activities towards MSM were at gay parties and gay sex venues. Male sex workers, MSM and female sex workers were also tested when they frequented the STI clinic on their own initiative.

### Study setting and population

Our STI clinic offers voluntary testing and treatment for STIs, covering the South Limburg region (population 600,000) with three STI test locations. Testing services are available free of cost to all high risk populations in the region. South Limburg is an urbanized area located in the south of the Netherlands, bordering to Germany and Belgium. The study population included all consultations by male sex workers, female sex workers and MSM who received STI and HIV testing and counselling at our clinic during the study period.

### Study procedures and definitions

At each new consultation, all clients were tested for STI and HIV. Specimens were collected in various ways: self-collected vaginal swabs (in female sex workers), urine (in male sex workers and MSM), anorectal swabs and oropharyngeal swabs; and, in a minority of cases, clinician-collected urethral and cervical swabs. Serum was drawn for serological testing. Specimens were processed at two regional laboratories using nucleic acid amplification assays (NAATs: strand displacement amplification: Becton Dickinson ProbeTec ET system, Sparks, MD, USA; and polymerase chain reaction (PCR): Roche Cobas Amplicor, San Francisco, CA, USA) for *Chlamydia trachomatis* (chlamydia) and *Neisseria gonorrhoeae* (gonorrhea). All positive gonorrhea samples were confirmed by an in-house PCR.

Serum was tested for *Treponema pallidum* (TPPA; MHA-TP; Fujirebio, Tokyo, Japan), rapid plasma reagin (Syfacard-R: Abbott Murex, Dartford, UK), fluorescent treponemal antibody absorption (Trepo Spt IF: BioMerieux SA, Marcy l’Etoile, France), hepatitis B (anti-Hbc, HbsAg, Axsym: Abbott Laboratories, Chicago, IL, USA) and HIV (anti-HIV1/2, Axsym: Abbott Laboratories, Chicago, IL, USA). Reactive samples were confirmed using Western blot (hivblot 2.2: Genelabs Diagnostics, Science Park, Singapore). All diagnostic tests were performed according to the manufacturers’ protocols. STI positivity was defined as a positive outcome on NAAT for chlamydia and/or a positive confirmation of gonorrhea, a new HIV infection, infectious syphilis (primary syphilis, secondary syphilis, or a recently acquired syphilis infection) and/or an infectious hepatitis B infection (HbsAg positive).

In addition to testing, trained STI nurses took a standardised medical and sexual history at each consultation, including data on sexual behaviour in the preceding six months and demographic data. All data were registered in an electronic patient registry and analysed with coded records. Free of cost treatment for chlamydia-infection, gonorrhea-infection and syphilis was offered at the STI clinic when appropriate, according to usual standards of care. Patients did not receive an incentive for testing. Patients who tested HIV positive were referred to the local HIV clinic.

Male sex workers were differentiated from MSM by means of self-identifying as working as male sex worker and being paid for providing internet escort services for men.

### Data analysis and ethical approval

The unit of analysis was a consultation. We compared demographic characteristics (age, ethnicity, hepatitis B vaccination status, positive history of STI in the last 2 years, known to be HIV positive) and sexual behaviour (number of sex partners, having sex with women, having had sex contact with a sex worker) of the male sex workers, female sex workers and MSM populations using *χ*2-tests (for categorical variables) and non-parametric tests (for continuous variables).

We conducted univariate and multivariate regression analyses using stepwise backward regression to evaluate the determinants (covariates) for STI positivity (outcome) in male sex workers compared to female sex workers and MSM (primary predictor). We considered a p-value <0.05 to be statistically significant. Variables that showed to test significant in univariate analyses were included in multivariate analyses. Analyses were performed using SPSS 21.0.0 (IBM Inc., Somers, NY, USA). Oral informed consent was provided by subjects. The Medical Ethical Committee of Maastricht University (identification number 11-4-108) approved the study.

## Results

### Characteristics of the study population

In total, 3716 consultations were included in analyses; 212 consultations among male sex workers, 801 among female sex workers and 2703 among MSM. The consultations involved 1901 persons (119 male sex workers, 371 female sex workers and 1411 MSM). Of the male sex workers, 63 % (75/119) had been in contact with our clinic only once during the 4 years of the study period as can be seen in Table 1. Of female sex workers and MSM, 32 and 35 % respectively contacted our clinic only once, while the others had multiple consultations. All male sex workers reported being engaged in internet escort work, street sex work was not reported. Paid sex contacts took place at the home or room of the male sex workers or his client. Fifty four percent of the male sex workers self-identified as heterosexual.

**Table 1:** Number of STI tests during the study period in male sex workers, female sex workers and MSM. Description of data: Number of consultations including STI tests in male sex workers, female sex workers and MSM during the study period

	Male sex workers	Female sex workers	Men who have sex with men
Number of STI tests in study period	n (%)	n (%)	n (%)
1	76 (64 %)	211 (57 %)	847 (60 %)
2	24 (20 %)	64 (17 %)	248 (18 %)
3	5 (4 %)	39 (11 %)	129 (9 %)
4	7 (6 %)	15 (4 %)	88 (6 %)
≥5	7 (6 %)	42 (11 %)	99 (7 %)
Total	119	371	1411

The consultations by male sex workers showed that male sex workers were younger (median age of 23 years) than female sex workers and MSM (both median age of 37 years, *p* < 0.01) and more often originated from Eastern European countries, especially Romania (*p* < 0.01), as shown in Table 2. In all consultations male sex workers reported sex with men in the preceding 6 months. Male sex workers reported sex with women as well, with over half reporting in the consultation that they had female sex partners compared to only 28 % of MSM and 15 % of female sex workers. In almost three quarters of consultations by male sex workers, a third of consultations by MSM, and nearly half of consultations by female sex workers, no full vaccination against hepatitis B was reported. The number of sex partners reported by MSM not engaged in sex work was lower than in female and male sex workers: MSM reported 1 to 3 recent sex partners in half of the consultations, and hardly reported more than 50 sex partners. In contrast, 50 or more sex partners were reported in 40 % of male sex workers’ and in 71 % of female sex workers’ consultations. Having sex contacts with other sex workers (paid or casual, non-paying sex contacts) was mentioned by one in six consultations of male sex workers, but hardly mentioned by MSM or female sex workers.

**Table 2:** Demographic and behavioural characteristics of consultations with male sex workers, female sex workers and MSM. Description of data: Demographic and behavioural characteristics of the male sex workers, female sex workers and MSM consultations

	Consultations with male sex workers	Consultations with female sex workers		Consultations with men who have sex with men (MSM)	
	*n* = 212	*n* = 801		*n* = 2703	
Age			^a^		^a^
Younger than 25 years	64 % (136)	13 % (104)		25 % (687)	
25 years and older	36 % (76)	87 % (697)		75 % (2016)	
Ethnicity			^a, b^		^a, b^
Eastern Europe	88 % (172)	23 % (144)		2 % (42)	
Europe (other)/ North America	4 % (8)	64 % (404)		92 % (2242)	
Africa/ Asia / Latin America	8 % (16)	14 % (86)		6 % (147)	
Unknown	(16)	(167)		(272)	
Having sex with men (%) in the past 6 months	100 % (212)	100 % (801)		100 % (2703)	
Having sex with women (%) in the past 6 months	58 % (123)	15 % (120)	^a^	28 % (753)	^a^
Number of sex partners in the past 6 months			^a^		^a^
1–3	13 % (26)	14 % (80)		47 % (1205)	
4–49	47 % (93)	15 % (87)		52 % (1342)	
≥50	40 % (80)	71 % (408)		1 % (38)	
Had sexual contact with a commercial sex worker in the past 6 months	17 % (36)	0 % (0)	^a^	3 % (75)	^a^
Hepatitis B vaccination status			^a^		^a^
Not vaccinated	26 % (54)	17 % (137)		15 % (404)	
Vaccination series not yet complete	45 % (96)	31 % (244)		20 % (531)	
Fully vaccinated	29 % (61)	52 % (412)		65 % (1713)	
Positive STI history in the past 2 years	42 % (88)	31 % (243)	^a^	44 % (1179)	
Known to be HIV positive	5 % (11)	1 % (7)	^a^	10 % (252)	^a^

^a^ Variable differs significantly (*p* < 0.05) between category and reference category male sex workers

^b^ Ethnicity is the only variable with few missing data (‘Ethnicity unknown’). All other characteristics have no missing data

### STI positivity

A new STI was diagnosed in 40 % of the consultations among male sex workers; this was significantly higher than in the consultations of female sex workers (4 %, *p* < 0.01) and MSM (14 %, *p* < 0.01) as shown in Tables 3 and 4.

**Table 3:** New diagnosed STI in consultations in male sex workers, female sex workers, MSM

	Consultations with male sex workers	Consultations with female sex workers	Consultations with men who have sex with men (MSM)
	*n* = 212	*n* = 801	*n* = 2703
*Chlamydia trachomatis*	18 % (39)	6 % (46)^a^	8 % (224)^a^
*Neisseria gonorrhoeae*	8 % (18)	3 % (23)^a^	5 % (70)^a^
Syphilis (infectious)	15 % (35)	1 % (4)^a^	1 % (48)^a^
Hepatitis B (infectious)	5 % (11)	0 % (1)^a^	0 % (7)^a^
HIV (new infection)	8 % (16)	0 % (3)^a^	1 % (24)^a^
Any new, infectious STI found during the consultation (*Chlamydia trachomatis, Neisseria gonorrhoeae*, syphilis, hepatitis B, HIV)	40 % (84)	9 % (73)^a^	14 % (383)^a^
More than one STI found during the consultation	12 % (25)	0 % (4)^a^	2 % (41)^a^

^a^ Variable differs statistically significantly (*p* < 0.05) compared to reference category

**Table 4:** Behavioural and demographic determinants associated with a newly diagnosed STI in male sex workers, female sex workers and MSM

	Univariate analysis	Multivariate analysis
	OR [95 %-CI]	OR [95 %-CI]
Category		
Male sex workers	ref	ref
Female sex workers	0.15 [0.11–0.22]^a^	0.27 [0.17–0.43]^a^
Men having sex with men (MSM)	0.25 [0.19–0.34]^a^	0.41 [0.26–0.66]^a^
Age		
Younger than 25 years	ref	ref
25 years and older	0.65 [0.54–0.80]^a^	0.68 [0.54–0.85]^a^
Ethnicity		
Europe (other)/ North America	ref	ref
Eastern Europe	2.46 [1.91–3.18]^a^	1.48 [0.99–2.22]
Africa/ Asia / Latin America	1.13 [0.79–1.62]	1.01 [0.69–1.48]
Having sex with women (%)	0.87 [0.70–1.07]	
Number of sex partners in the past 6 months		
1–3	ref	ref
4–49	1.48 [1.21–1.80]^a^	1.30 [1.05–1.61]^a^
50+	1.37 [1.04–1.80]^a^	1.42 [0.99–2.04]
Had sexual contact with a commercial sex worker in the past six months		
Did not engage in sex contact with commercial sex worker in the past 6 months a commercial sex worker in the past six months	ref	
Did have sexual contact (paid or non-paying) with	0.77 [0.43–1.39]	
Hepatitis B vaccination status		
Not vaccinated	ref	
Vaccination series not yet complete	1.14 [0.85–1.53]	
Fully vaccinated	0.98 [0.76–1.27]	
Positive STI history in the past 2 years		
No previous STI history in the past 2 years	ref	
Positive STI history in the past 2 years	1,73 [1,44–2,08]^a^	1.53 [1.25–1.88]^a^
Known to be HIV positive		
Not known to be HIV positive	ref	
Known to be HIV positive	2.82 [2.14–3.73]^a^	2.53 [1.86–3.43]^a^
Sequence of test during the study period		
First STI test	ref	
Second STI test	0,96 [0.76 – 1.22]	
Third STI test	0,65 [0,47 – 0,90]	
Fourth STI test	0,90 [0,62 – 1,31]	
Fifth and following STI tests	1,03 [0,75 – 1,40]	

^a^ Variable differs statistically significantly (*p* < 0.05) compared to reference category

Chlamydia was the most frequently detected STI. The prevalence of chlamydia, gonorrhea, HIV, infectious hepatitis B and infectious syphilis was highest in male sex workers compared to female sex workers (*p* < 0.01) and MSM (*p* < 0.05). Male sex workers were also significantly more likely to test positive for multiple STI than MSM or female sex workers (*p* < 0.01).

### Determinants of STI positivity

Being a male sex worker (compared to MSM and female sex workers) was univariately associated with the risk of being tested positive for an STI. Male sex workers tested 6.5 times more often positive for STI in a consultation than female sex workers (*p* < 0.01) and 4 times more often than MSM (*p* > 0.01). Other risk factors as age (categorical), ethnicity (categorical), number of sex partners in the last six months (categorical), history of previous STI (binomial) and known HIV positivity (binomial) were all univariately significantly associated with STI positivity, as shown in Table 4. When in multivariate analyses correcting for these covariates, the higher risk of being positive for STI among male sex workers compared to female sex workers (OR 3.7, *p* < 0.01) and MSM (OR 2.4, *p* < 0.01) remained, although risk estimates somewhat attenuated.

To analyse possible bias caused by double enrolment, analyses were repeated on individual level. In patients who were enrolled multiple times, only the results of their most recent consultation were used. This did not significantly influence the descriptive statistics nor the STI positivity in male sex workers. Repeating the regression analyses on individual level, an even stronger association was found between STI positivity and being a male sex workers compared to female sex workers (OR 6.0, *p* < 0.01, after correction for age, STI history and known HIV positivity. Ethnicity and number of sex partners did not show significant in these analyses) and MSM (corrected OR 5.3, *p* < 0.01).

## Discussion

We present a large study on male sex workers and compared their characteristics to those of two established key populations for STIs: female sex workers and other MSM served by the same STI clinic. Male sex workers were at a very high risk of contracting STIs and HIV: in more than 40 % of STI and HIV tests in male sex workers a new infection was diagnosed. Male sex workers were diagnosed with a new STI (including HIV) during a consultation 6.5 (95 %-CI: 4.5–9.4) times more often than female sex workers and 4.0 (95 %-CI: 3.0–5.3) times more often than MSM. Male sex workers remained at greater risk even after correction for behavioural and demographic differences between the 3 groups, suggesting that being a male sex workers is a risk factor in itself and that specific interventions need to be targeted at male sex workers. Male sex workers form a hidden key population for STI control and, since they are hard to reach and largely invisible to regular care, European public health authorities should intensify efforts to offer them STI testing, treatment and prevention. We have demonstrated that it is difficult, but feasible to reach out to this high risk population.

The sexual contacts of male sex workers in our study were not limited to men or male customers. Male sex workers also reported to have sex with women and with other sex workers, making them a potential bridging population for STI and HIV. In internet escort services, safe sex aspects (i.e. proper use of condoms and lubricants) are often not negotiated [20]. Previous studies also found that male sex workers to be at a high risk for contracting and spreading STI and HIV, also towards the heterosexual population [5, 13, 14, 21]. A risk reduction strategy for male sex workers is therefore urgently needed. Not only do male sex workers have high STI and HIV positivity, but they exhibit high sexual risk behaviour and the potential to transmit STI: to their anonymous customers, and also outside this risk population. Interventions aimed at female sex workers and MSM will not reach the male sex workers population, as they tend not to self-identify as sex workers or, nor as homosexual or bisexual. This stresses the need to invest in targeted interventions.

Previous studies in European countries found HIV positivity in male sex workers ranging from 1 to 12 % [5, 8, 13–15]. Other STIs were also frequently detected, with positivity ranging between 6 % [4] and 46 % [14]. The male sex workers in our study show to be at the high end of this range. A selection bias might have taken place: possibly male sex workers more at risk were more likely to attend STI and HIV testing. Low hepatitis B vaccination grades were consistent with those found in other studies [22]. No previous comparison of STI rates with both female sex workers and MSM in the same setting had been made; our results demonstrate that male sex workers are a distinct population. The differences in age and ethnic origins between male sex workers and female sex workers and MSM, but also the internet-oriented status of their sex work, makes that male sex workers require a unique approach.

We expect this study sample to represent internet-based male sex workers, MSM and female sex workers in the Netherlands, although local differences might limit the generalizability of our results for all countries in Europe. Nevertheless, as this subculture is based on money-driven internet activities that are not limited by borders within the European Union, we expect male sex workers to be active in many European countries. The sampling methods in male sex workers included snowball sampling. This tends to concentrate the sample within a particular group, which could explain the fact that male sex workers were largely from a particular age group and ethnic background. This also may have biased our results towards one particular high risk group. Another potential source of bias in our data is that male sex workers included in this study have been self-selected: probabilistic sampling of male sex workers was not feasible and underreporting of engagement in sex work may have taken place. If many MSM would not report engaging in sex work with men, the differences in STI positivity between MSM and male sex workers may actually be larger. Face-to-face data collection may also have induced underreporting of behavioural risk factors, by replying with ‘desirable responses’. If cultural differences would be playing a part in this underreporting, this could be a possible source of bias. By using clinical-based data, clients that are more at risk may have attended our clinic more frequently. As a consequence of the analysis on the consultation level, multiple consultations with the same person could be a source of confounding. This however showed not to be significantly related to STI positivity in univariate analysis. Repeating regression analyses on individual level, an even stronger association was found between STI positivity and being a male sex workers compared to female sex workers and MSM. In our analysis we have made a comparison between internet-based male sex workers and MSM and female sex workers that were not having only internet-based contacts. Possibly subpopulations of internet-based female sex workers and MSM who meet their (sex) partners predominantly online also are at a higher risk for STI and HIV. This should be addressed in future studies.

The residency of male sex workers in the Netherlands is characterised by impermanence and unpredictability. It was often mentioned to us by the male sex workers that they frequently travel between, and migrate to other European countries, increasing our concern for their potential to enhance STI transmission between countries. As 95 % of our region shares a border with Germany and Belgium, cross-border traffic was common within the male sex workers population in our study, increasing the urgency for cross-border policy. The regular organisation of health care does not accommodate for brief contact moments like these, making male sex workers a hard population to engage in long-term care. This increases the risks of disease transmission throughout Europe. Public health interventions aimed at targeting male sex workers should allow for impermanent residency and should include (inter)national cooperation. The internet offers chances for male sex workers to easily connect to potential clients, but it also has the opportunity to connect to male sex workers to provide a safe sex message, counselling on sexual behaviour and STI testing [23]. So far no studies have been conducted about how long male sex workers sustain their high-risk behaviour [24] or about the migration patterns of male sex workers; such patterns should be addressed in future research.

## Conclusions

In conclusion, internet-based male sex workers having sex with men demonstrate high positivity of STI including HIV. Yet these men are not reached by regular health care interventions aimed at female sex workers and MSM. These hard-to-reach male sex workers do not limit their sex contacts to male customers only and have sex with women and co-workers as well. Therefore, male sex workers may form a bridge population in HIV and STI transmission. With intensive, proactive outreach strategies, we were able to reach out to the male sex workers population and deliver health care to this elusive key population, which is urgently in need of targeted interventions and health care.

## Abbreviations

Chlamydia: Infection with *Chlamydia trachomatis*
Gonorrhea: Infection with *Neisseria gonorrhoeae*
HIV: Human immunodeficiency virus
MSM: Men who have sex with men
NAAT: Nucleic acid amplification assay
PCR: Polymerase chain reaction
STI: Sexually transmitted infections
